# Application of polyglycolic acid sheets and basic fibroblast growth factor to prevent esophageal stricture after endoscopic submucosal dissection in pigs

**DOI:** 10.1007/s00535-023-02032-4

**Published:** 2023-08-27

**Authors:** Yusuke Nishimura, Masayoshi Ono, Naoto Okubo, Takayuki Sone, Masayuki Higashino, Shogo Matsumoto, Marina Kubo, Keiko Yamamoto, Shoko Ono, Shunsuke Ohnishi, Naoya Sakamoto

**Affiliations:** 1 Department of Gastroenterology and Hepatology, Hokkaido University Graduate School of Medicine, Kita-14-Jo Nishi-5-Chome Kita-Ku Sapporo, Hokkaido, 060-8648 Japan; 2 Division of Endoscopy, Hokkaido University Hospital, Sapporo, Japan; 3 Laboratory of Molecular and Cellular Medicine, Faculty of Pharmaceutical Sciences, Hokkaido University, Sapporo, Japan

**Keywords:** Basic fibroblast growth factor, Polyglycolic acid, Esophageal stricture, Endoscopic submucosal dissection

## Abstract

**Background:**

Endoscopic submucosal dissection (ESD) has been the first-line treatment for early-stage esophageal cancer. However, it often causes postoperative stricture in cases requiring wide dissection. Basic fibroblast growth factor (bFGF) reportedly has anti-scarring effects during cutaneous wound healing. We hypothesized that suppressing myofibroblast activation will prevent stricture after esophageal ESD.

**Methods:**

We resected a complete porcine esophagus circumference section by ESD. To investigate the preventive effect of bFGF on esophageal stricture formation after ESD, we endoscopically applied bFGF-soaked poly-glycolic acid (PGA) sheets onto the wound bed after ESD and fixed them by spraying fibrin glue (PGA + bFGF group), PGA sheets alone onto the wound bed and fixed them by spraying fibrin glue (PGA group), or nothing (control group). After removing the esophagus on day 22, we evaluated the mucosal constriction rate.

**Results:**

Compared with those in the control group, esophageal stricture was significantly reduced in the PGA + bFGF group, and the areas stained with α-SMA and calponin-1 antibodies were significantly inhibited in the PGA + bFGF and PGA groups. The thickness of the fibrous layer in the PGA + bFGF group was uniform compared to that of the other groups. Thus, PGA + bFGF inhibited the development of unregulated fibroblasts in the acute phase, leading to uniform wound healing.

**Conclusions:**

Stenosis after esophageal ESD is related to fibrosis in the acute phase. Administration of PGA and bFGF suppresses myofibroblast activation in the acute phase, thereby preventing esophageal constriction in pigs.

**Supplementary Information:**

The online version contains supplementary material available at 10.1007/s00535-023-02032-4.

## Introduction

Esophageal cancer (EC) is the ninth most common cancer worldwide, accounting for approximately 5.3% of all cancer-related mortality [1]. Its treatment options include endoscopic resection, chemotherapy, surgery, and radiotherapy. With the development of endoscopic technology, the number of early-stage cases detected has increased [2, 3].

Endoscopic submucosal dissection (ESD) for gastrointestinal tumors may cause adverse events such as perforation, but en bloc resection is possible even for tumors larger than 20 mm in any location [4, 5]. In conventional endoscopic mucosal resection (EMR), the major concern is local recurrence, with a prevalence as high as 20% [6]. Takahashi et al. reported that the local recurrence rate of superficial EC was 9.8% after EMR and only 0.9% after ESD [7]. Therefore, ESD is widely accepted as the first-line treatment for early-stage EC, especially for endoscopic resection [8]. However, stenosis risk is extremely high, reaching up to 66–100%, when a circumferential mucosal defect involves over three-fourth of the esophageal circumference. Multiple balloon dilation sessions are occasionally required, lowering patients’ quality of life [9–13]. After ESD, inflammation and fibrosis also occur in the esophagus, reducing its elasticity and compliance, ultimately causing postoperative stricture [14].

Typically, balloon dilation, local injection of steroids, or oral steroid administration is used to prevent strictures after ESD. Although these methods are effective, adverse events, such as perforation, mediastinum abscess, and steroid-induced side effects, might occur [15–17]. Furthermore, prophylactic steroid administration is reportedly effective in preventing stenosis of mucosal defects in 7/8 circumferences of esophagus, but not in all-circumferential defects [18]. Therefore, new preventive measures are needed for defects that affect the entire circumference ESD of the esophagus.

The application of poly-glycolic acid (PGA) sheets combined with fibrin adhesive has also been reported to prevent stricture after esophageal ESD [19]. Currently, the mechanism by which PGA sheets prevent stenosis remains unclear, but using fibrin adhesives alone cannot effectively prevent stenosis [20]. Hence, the combination of PGA sheets and fibrin glue may have a synergistic effect. However, Sakaguchi et al. reported that PGA sheets alone are insufficient and that combining PGA sheets with steroid injection is more effective [21]. Therefore, a simple method should be developed that is less harmful than steroids and not prone to adverse events, such as perforation, infection susceptibility, and worsening of diabetes.

Fibroblast growth factors (FGFs) constitute a large family of signaling polypeptides that are expressed in various cell types from early embryogenesis to adulthood [22]. Some FGFs appear only in embryonic tissues, whereas other FGFs, such as basic FGF (bFGF or FGF2), appear in both embryonic and adult tissues. In adult tissues, FGF promotes wound healing [23–26]. In particular, bFGF induces cell proliferation and differentiation, promoting migration, resulting in an angiogenic effect [27]. It is a potent mitogen and chemoattractant for endothelial cells, fibroblasts, and keratinocytes. Additionally, it stimulates metabolism, growth of the extracellular matrix, and movement of mesodermal-derived cells [28]. In 2001, Kaken Pharmaceutical Co., Ltd. (Tokyo, Japan) released Fiblast®Spray, a recombinant human bFGF (rhbFGF) preparation, as a topical spray. Presently, this spray is widely used for wound healing and scar suppression in clinical settings in Japan [29]. The administration of recombinant bFGF to skin wounds can accelerate acute and chronic wound healing [30–33]. In addition to suppressing skin scar contraction [34], bFGF is effective in inducing bronchodilation in rabbits [35]. Thus, it may also be effective in preventing luminal stenosis in the gastrointestinal tract. However, its use in gastrointestinal tract treatment remains unreported, and its efficacy in preventing stricture after esophageal ESD is unknown. Hence, this study aimed to evaluate the effectiveness of the combination of PGA sheets and bFGF through a comparative analysis.

## Methods

### Animals

This study used female domestic pigs (20–25 kg; Sankyo Labo Service, Tokyo, Japan), and the Animal Care and Use Committee of Hokkaido University approved our experimental protocol and have therefore been performed in accordance with the ethical standards laid down in the 1964 Declaration of Helsinki and its later amendments.

### Animal model

First, we injected the pigs with atropine (1 mg; Terumo, Tokyo, Japan) intramuscularly and waited for 10 min. Then, anesthesia was administered to nine pigs by the intramuscular injection of midazolam (1.6 mg; Astellas, Tokyo, Japan), butorphanol (0.8 mg; Meiji Seika Pharma, Tokyo, Japan), and dexmedetomidine hydrochloride (0.4 mg; Nippon Zenyaku Kogyo, Fukushima, Japan). Subsequently, these pigs were intubated and connected to a mechanical ventilator under 3% sevoflurane in oxygen. While ESD was performed, the heart rate, 3-lead electrocardiography, and peripheral oxygen saturation (Nihon Kohden, Tokyo, Japan) were continuously monitored. This procedure used a single-channel gastrointestinal endoscope (GIF-Q240; Olympus, Tokyo, Japan) with a transparent attachment hood fitted to the tip (Top, Tokyo, Japan).

Using Dual knife J (Olympus, Tokyo, Japan), an incision line was marked on the lower part of the esophagus 39–42 cm from the incisors while looking at the scale on the scope for the complete circumference and 3 cm from the long axis. This device has a small non-insulated dome-shaped electrode on the tip of the 1.5 mm knife and possesses a water supply function. Furthermore, we used a 25G needle (Top) to inject a glycerol solution into the submucosal layer. After injection, we used Dual knife J to achieve a circumferential incision. Then, an electrosurgical generator (ESG-100; Olympus) was set to the pulse cut slow mode (40 W) or forced coagulation mode (50 W) for mucosal and submucosal incisions. Hemorrhage was controlled using hemostatic forceps, such as the Coagrasper (Olympus), in the soft coagulation mode (40 W). All ESD procedures were performed by one endoscopist (YN). In the groups using PGA sheets (Neoveil® sheet; GUNZE, Kyoto, Japan), the sheets were applied immediately after the ESD, and information, such as the duration of the procedure and the number of PGA sheets, was recorded. For postoperative care, all pigs were fed with liquids on the day after ESD, followed by solids in the subsequent days.

### Experimental design

To evaluate the preventive effect of bFGF on esophageal stricture formation after ESD, we divided the pigs into three groups: the bFGF-soaked PGA sheet group (PGA + bFGF group), the PGA sheet group (PGA group), and the control group, with three pigs per group. Approximately 20 PGA sheets cut into 1 cm^2^ were prepared and applied to the wound. The sheets were fixed in place by spraying fibrin glue in the PGA group. In the PGA + bFGF group, the PGA sheets were soaked in bFGF (250 μg/2.5 mL) before application; each 1-cm^2^ PGA sheet was soaked in Fiblast Spray (0.8 mL) containing bFGF (80 μg), and was fixed in place by spraying fibrin glue after application. Assuming that the diameter of the esophagus is 2 cm and the circumference is a little over 3 cm, the ESD wound area of 3 cm is about 20 cm^2^, so 20 sheets were prepared. All 20 PGA sheets were used as much as possible in each pig. Conversely, the control group received nothing after ESD. On day 8, we assessed the esophagus using an endoscope and evaluated the state of the wound and the degree of stricture. During this, balloon dilation and bougie were not performed, and only observation was performed.

### Assessment of the degree of esophageal stricture after ESD

On day 22, the pigs were killed by an intravenous injection of 20 mL of 15% potassium chloride (Terumo) after general anesthesia. We incised the anterior neck and abdomen and removed the esophagus transactionally. The resected esophagus was immediately placed on a rubber board and fixed with pins. The degree of stricture at the lesion site was expressed as the lateral mucosal constriction rate, calculated by the following formula, as described previously [11, 36]:

Mucosal constriction rate (%) = [1 − (length of the short axis at the site of maximal constriction)/(length of the short axis at a normal mucosal site on the upper side + length of the short axis at a normal mucosal site on a lower side)/2] × 100.

The length was measured using a ruler with a scale of 1 mm.

### Histologic and immunohistochemical examination

The esophagus was fixed in 40 g/L formaldehyde saline solution, embedded in paraffin, and cut into 5-mm sections. Sections were made along the minor axis of the esophagus. Tissue sections underwent hematoxylin and eosin staining and immunostaining to assess the condition of the wound tissue. For the immunostaining, we used anti-α-smooth muscle actin (α-SMA) antibody (clone 1A4, 1:1000 dilution; Sigma-Aldrich), anti-calponin-1 (CNN1) antibody (1:1000 dilution; Abcam, Cambridge, UK), anti-myeloperoxidase (MPO) antibody (1:300 dilution; Thermo Scientific, Waltham, Mass), and anti-CD107a antibody (clone 4E9/11, 1:300 dilution; AbD Serotec, Kidlington, UK). We photographed five fields per section from each pig and measured the stained area per field of view (× 200, scale bar: 50 μm) with a digital image analyzer (WinROOF 2018; Mitani Co., Fukui, Japan). Moreover, collagen fibers were stained with Elastica–Masson staining. We randomly selected five locations, including the thickest and thinnest stained fiber layers, and measured the thickness using cellSens (Olympus, Tokyo, Japan) at × 40 (scale bar: 200 μm). To evaluate the state of tissue regeneration in the entire wound, we measured not only the fibrous layer but also the thickness from the bottom edge of the area stained with Elastica–Masson staining to the surface layer of the wound.

### Statistical analysis

Given that our study is the first to assess the effect of dilation on esophageal stenosis in pigs, we had no data for calculating the sample size in advance. Therefore, we first aimed to perform an experiment using nine pigs and a post hoc power analysis to verify the sample size. We estimated that using nine pigs was sufficient. Data are expressed as mean (standard deviation). Parameters among the groups were compared by one-way analysis of variance followed by unpaired Student’s *t*-test and two-tail test. Differences were considered statistically significant if *P* < 0.05. All statistical data were analyzed using GraphPad Prism version 8 (GraphPad Software, CA, USA).

## Results

### Characterization of ESD

**Fig. 1:**
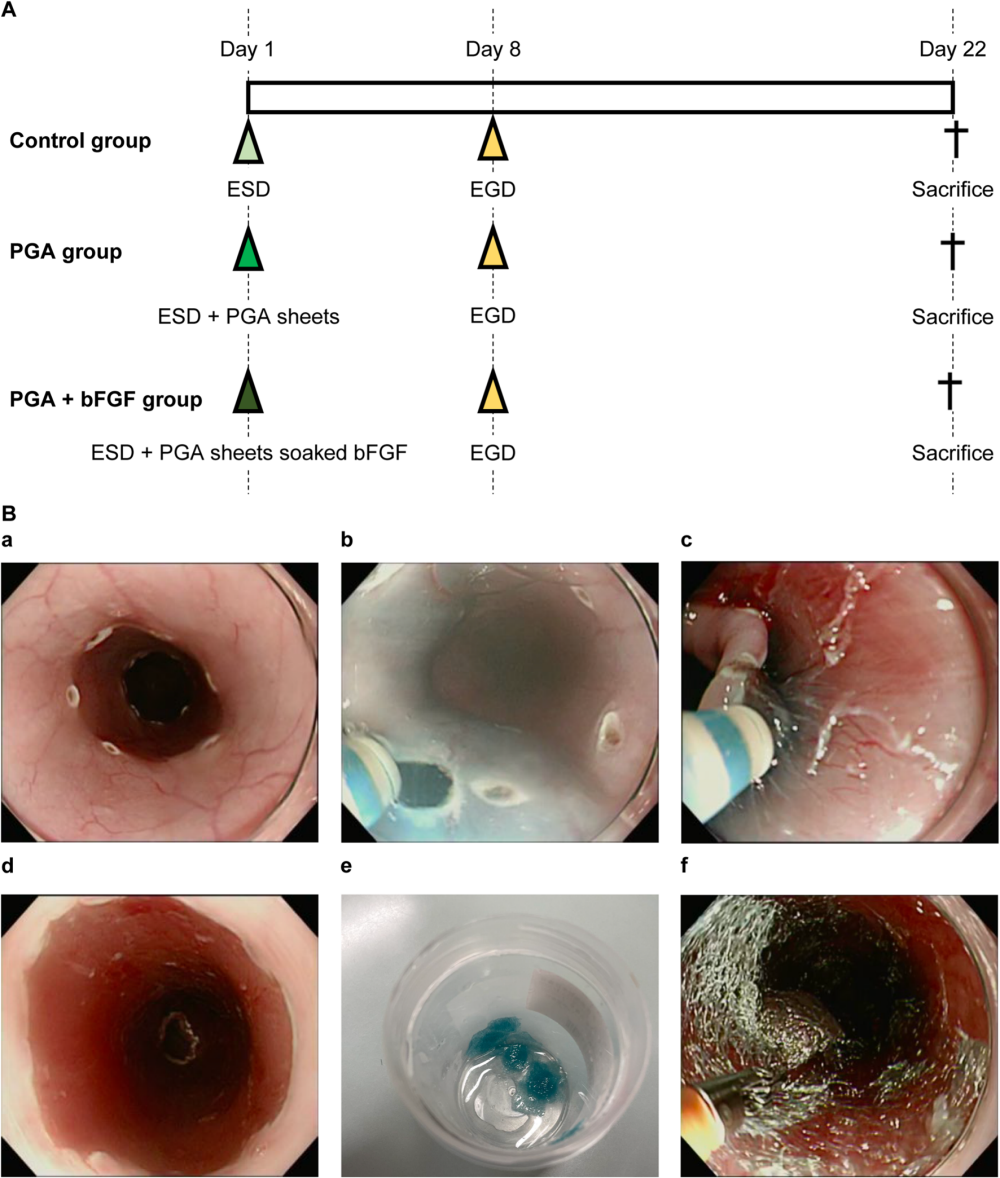
Experimental protocol for the esophageal model. **A** Experimental protocol for the ESD model. ESD was performed on all pigs on day 1. For the PGA group, 1 cm^2^ PGA sheets were delivered to the wound and fixed by spraying fibrin glue. For the PGA + bFGF group, 1 cm^2^ PGA sheets soaked with bFGF were delivered to the wound and fixed by spraying fibrin glue. **B** The ESD procedure was performed as follows: **a** the incision line was marked using Dual knife J on the lower part of the esophagus in a complete circumference and 3 cm of the long axis; **b** the mucosa was cut circumferentially; **c** the submucosa was dissected; **d** ESD was completed; **e** the PGA sheet cut into 1 cm^2^ was soaked with bFGF;** f** the PGA sheets were applied. ESD, endoscopic submucosal dissection; bFGF, basic fibroblast growth factor; PGA, polyglycolic acid

**Fig. 2:**
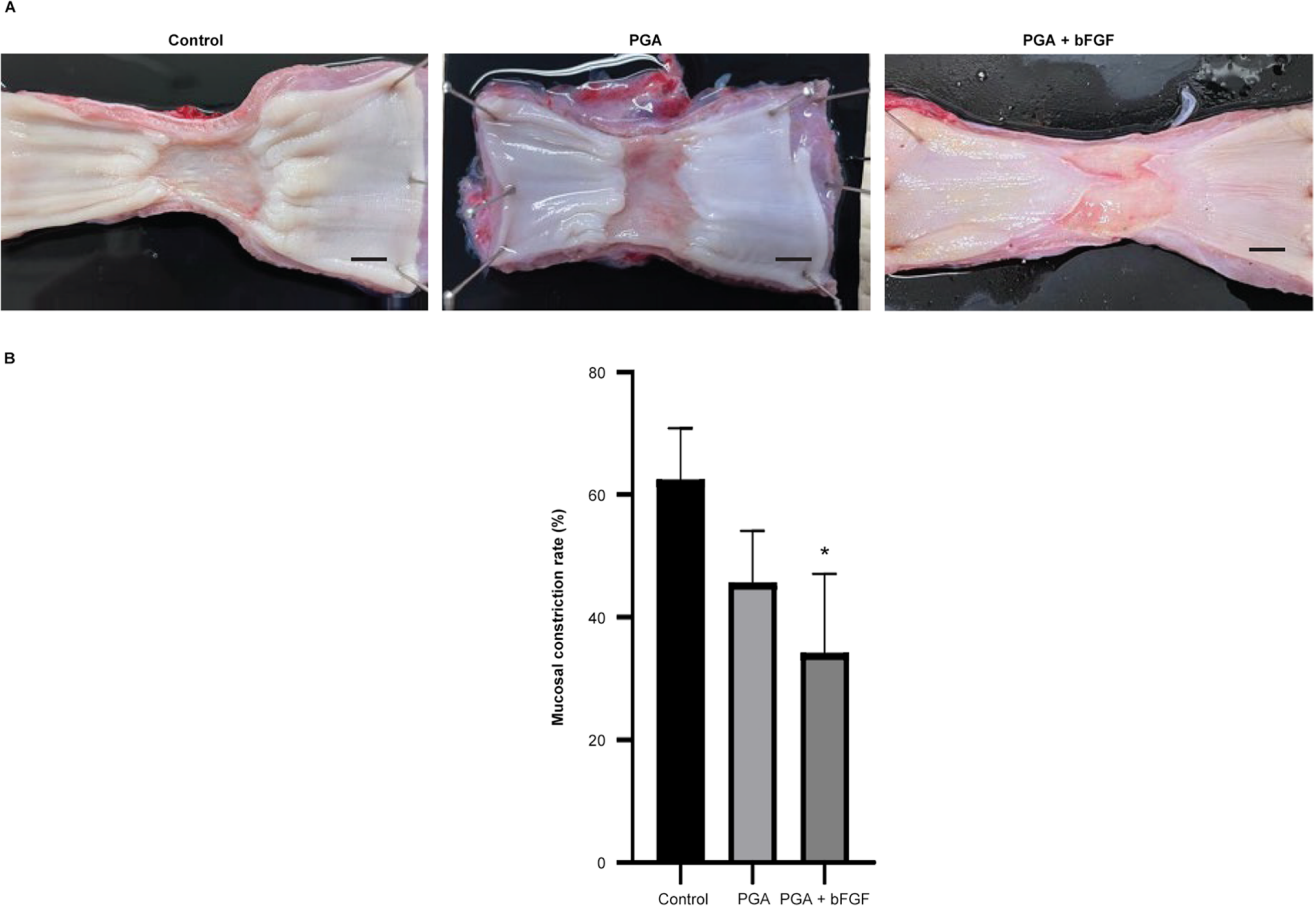
Preventive effects of bFGF on esophageal strictures 3 weeks after endoscopic submucosal dissection. **A** Macroscopic image of the esophagus. Scale bars, 10 mm. **B** Mucosal constriction rate. Values are expressed as the mean (standard deviation) of three animals per group. *P < 0.05 vs. control group. The control group received no treatment after ESD. In the PGA group, PGA sheets were attached to the wound and sealed with fibrin glue. In the PGA + bFGF group, PGA sheets soaked with bFGF were attached to the wound and sealed with fibrin glue. Three weeks after endoscopic submucosal dissection, bFGF prevented the occurrence of esophageal stricture. *bFGF* basic fibroblast growth factor, *PGA* polyglycolic acid

Figure 1 depicts the experimental protocol. On day 8, stenosis had begun in the control group and passage through the scope was impossible. Although there was mild stenosis in the PGA group, the scope having a maximum diameter of 11.9 mm passed without resistance, and the scope the PGA + bFGF group passed through the diameter margin. On day 22, esophageal stricture was significantly suppressed in the PGA and PGA + bFGF groups compared with that in the control group (control: 63.6% [8.4], PGA: 42.2% [8.4], PGA + bFGF: 36.7% [12.9]) (Fig. 2).

### Histologic analysis of the esophagus after applying bFGF

**Fig. 3:**
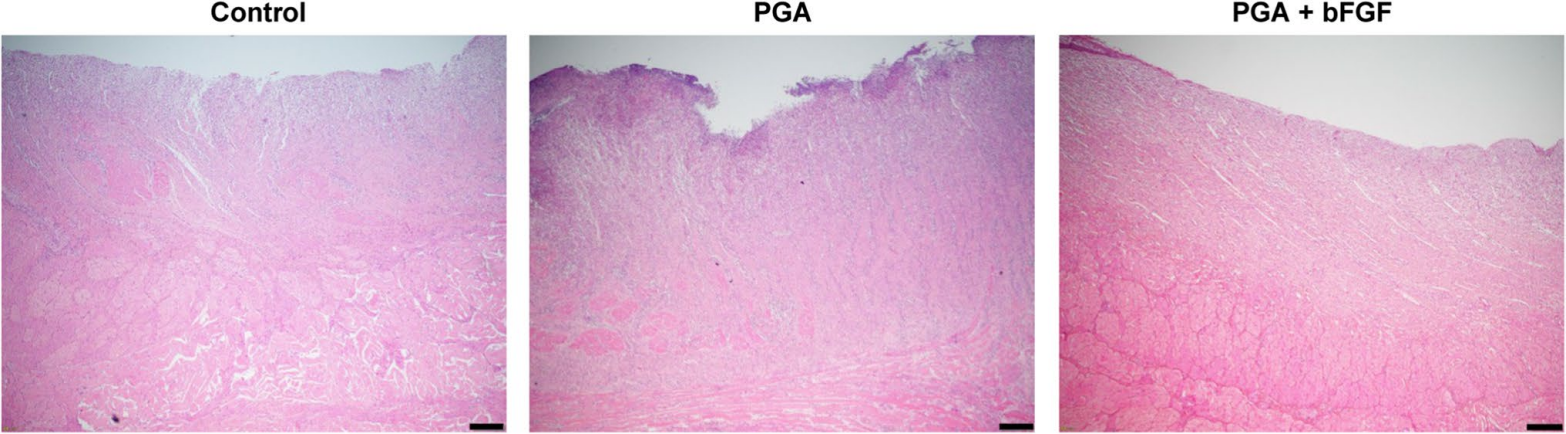
Effect of bFGF on histologic findings 3 weeks after endoscopic submucosal dissection. Evaluation by hematoxylin and eosin staining. Scale bar, 200 μm. *bFGF* basic fibroblast growth factor, *PGA* polyglycolic acid

We investigated the pathological effect of bFGF in a porcine esophageal ESD model. Hematoxylin and eosin staining demonstrated that the regenerated tissue arrangement was more ordered in the intervention groups than in the control group, and this tendency was more pronounced in the PGA + bFGF group than in the PGA group (Fig. 3).

The expression rate of MPO-positive neutrophils was not significantly different between the PGA + bFGF, PGA, and control groups (6.1%, 9.5%, and 8.6%, respectively). The expression rate of CD107a-positive macrophages also showed no significant difference (4.3%, 4.1%, and 4.0%, respectively). Thus, PGA or bFGF administration did not change the inflammatory response or immune system activation (Supplemental Fig. 1).

**Fig. 4:**
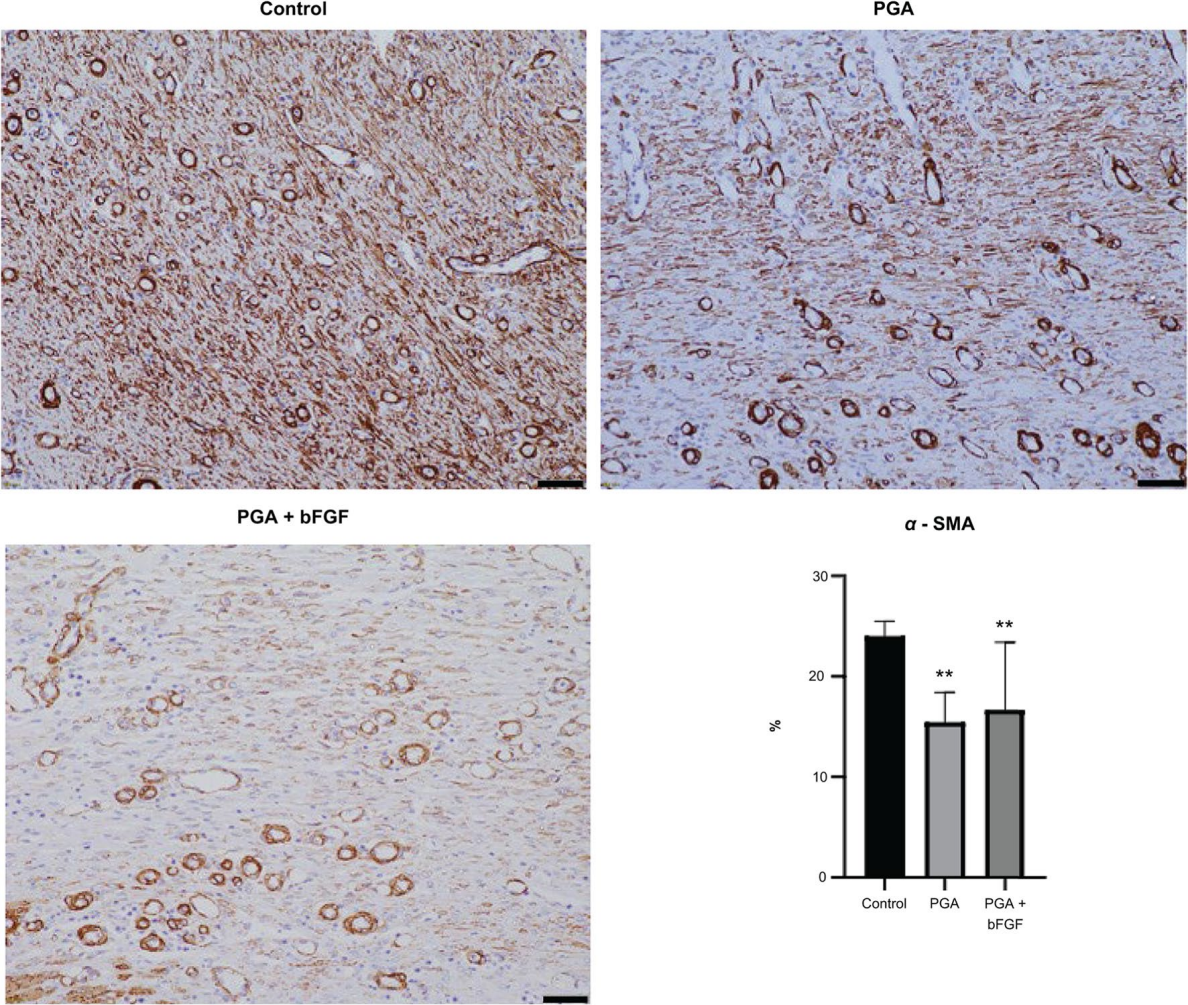
Expression of α-SMA. Scale bar, 50 μm. Values are expressed as means ± standard deviation (SD). **P < 0.01 vs. control group α-SMA, smooth muscle actin; *bFGF* basic fibroblast growth factor, *PGA* polyglycolic acid

**Fig. 5:**
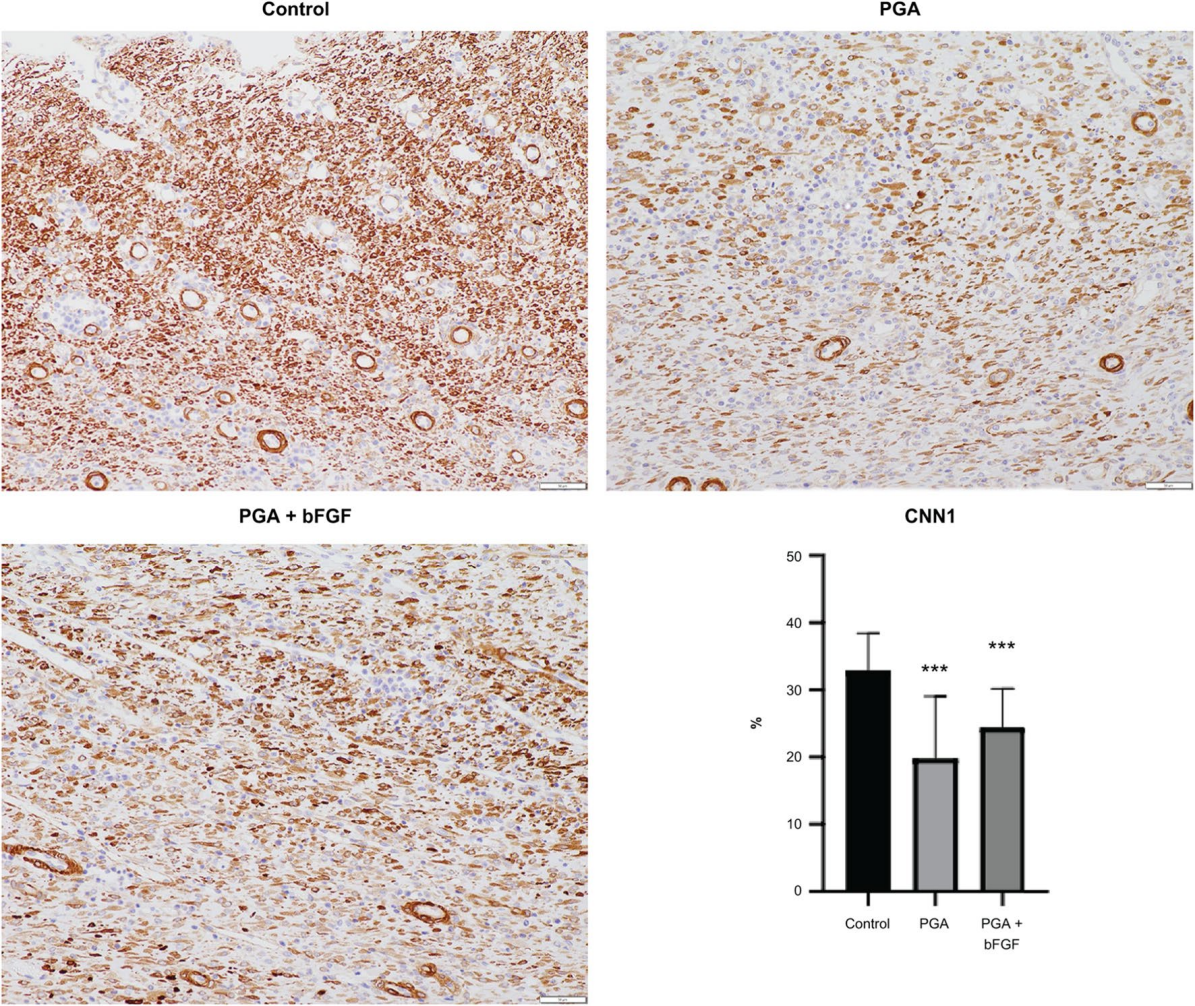
Expression of calponin-1 (CNN1). Scale bar, 50 μm. Values are expressed as means ± standard deviation (SD). ***P < 0.001 vs. control group. bFGF, basic fibroblast growth factor; CNN1, calponin-1; PGA, polyglycolic acid

Moreover, areas stained with anti-α SMA antibody were significantly less in the PGA + bFGF and PGA groups compared with those in the control group (PGA + bFGF: 16.7%, PGA: 15.5%, control: 24.0%) (Fig. 4). Significant suppression in areas stained with anti-calponin-1 antibody was also observed in the PGA + bFGF and PGA groups compared with those in the control group (24.4%, 19.8%, and 32.9%, respectively) (Fig. 5). Elastica–Masson staining showed that ESD caused disorganized, loose fibrous changes, with fibrous accumulation extending to the muscularis propria. However, when bFGF-soaked PGA sheets were applied, the heterogeneity of fibrosis was significantly reduced.

**Fig. 6:**
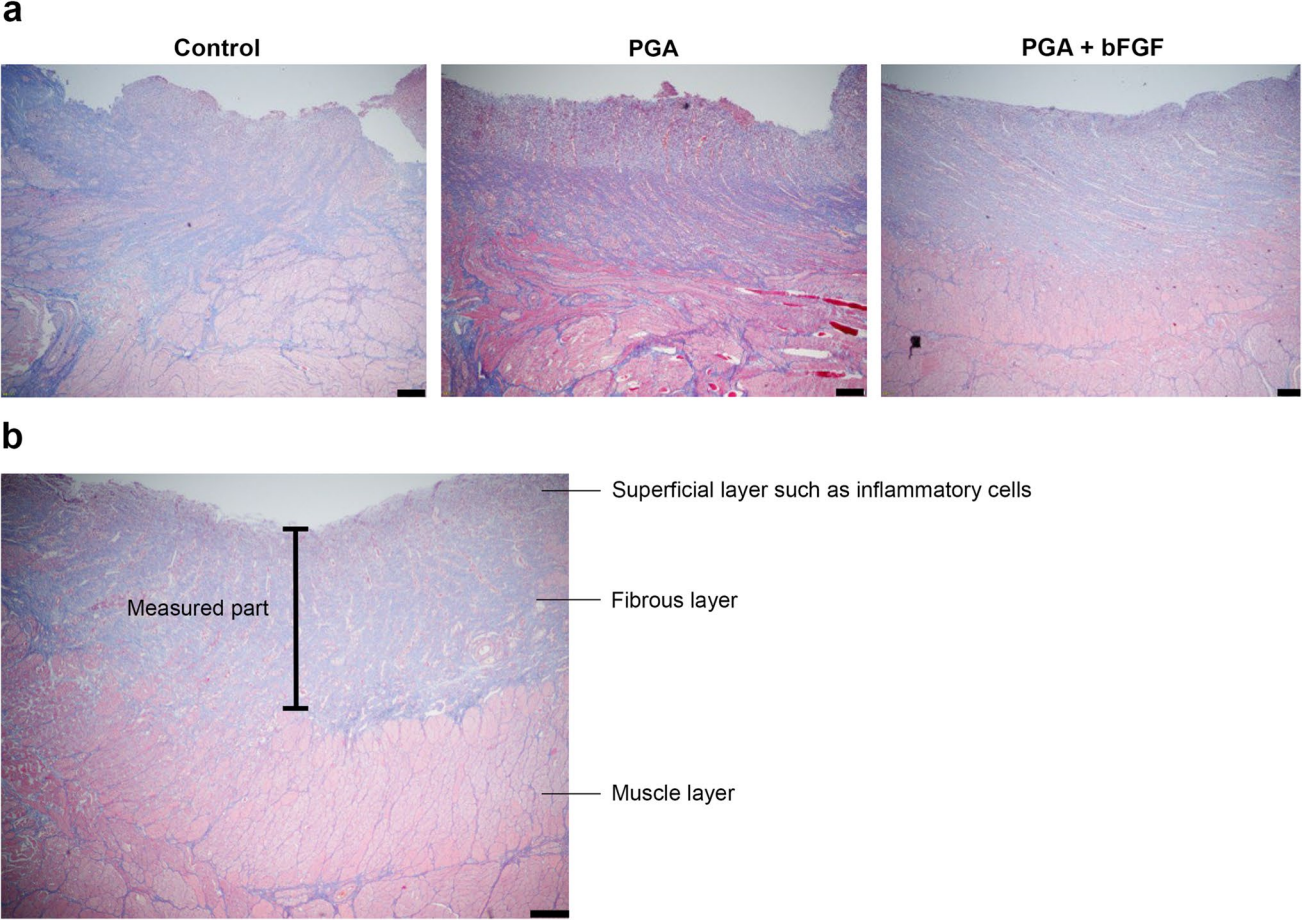
Elastica–Masson staining and the fiber thickness. **A** Elastica–Masson staining. Scale bar, 200 μm. **B** Definition of measurement method. (1) Thickness of the fibrous layer alone; (2) thickness from the bottom edge of the area stained with Elastica–Masson staining (the fibrous layer) to the surface layer of the wound. Scale bar, 200 μm

When measured to the superficial layer of the wound to assess the uniformity of fibrosis, the difference between the thinnest and thickest areas was the smallest in the PGA + bFGF group compared with the PGA and control groups (467.3, 934.9, and 726.2 μm, respectively). Therefore, the PGA + bFGF group had uniform fibrosis. Furthermore, the mean of five measured locations was thicker in the PGA + bFGF and PGA groups than in the control group (1131.3, 1358.1, and 963.0 μm, respectively). The fibrosis in the control group was thin and disordered, whereas that in the PGA + bFGF group was thick and orderly (Table 1, Fig. 6).

**Table 1:** The evaluation of the thickness of the fibrosis layer

	Control	PGA	PGA + bFGF	Analysis of variance (*P* value)
A µm (SD)	963.0 (115.4)	1358.1 (440.7)	1131.3 (151.3)	*P* = 0.20
B µm (SD)	726.2 (182.6)	934.9 (59.0)	467.3 (174.5)	*P* = 0.02

Control, control group receiving no treatment; PGA, PGA group receiving 1 cm^2^ PGA sheets alone; PGA + bFGF, PGA + bFGF group receiving PGA sheets soaked with bFGF

A: Mean value of the thickness of the fibrosis layer: thickness from the lower end of the fibrous layer to the surface layer of the wound

B: Difference between the thinnest and thickest areas

The only significant difference between the PGA and PGA + bFGF groups was fibril homogeneity. However, this suggests that the simple method of impregnating with bFGF promotes clean healing. Attaching the PGA sheet is also time-consuming, but the benefit is greater than the cost incurred when severe stenosis occurs.

## Discussion

This study investigated whether bFGF can effectively prevent esophageal stricture formation after ESD. The results are as follows: (1) the PGA sheet saturated with bFGF prevented esophageal stenosis; (2) bFGF inhibited myofibroblast activation in the esophagus; and (3) bFGF suppressed acute unregulated fibrosis and promoted normal wound healing.

In cutaneous wounds, bFGF reportedly exhibits anti-scarring effects during wound healing [37, 38]. Therefore, we hypothesized that suppressing myofibroblast activation can prevent stricture after esophageal ESD. Currently, bFGF is marketed as Fiblast Spray and is easy to procure. Given that it is a liquid, it can be delivered to the wound by simply soaking the PGA sheet; thus, the delivery method is not complicated.

PGA sheets have already been reported to be effective in preventing stricture in humans [19, 39, 40]. We have shown for the first time that PGA is also effective in preventing stricture in pigs. Our study also confirmed that PGA sheets prevent moderate stenosis and that adding bFGF can further prevent stenosis. The PGA sheets covered the wound and protected it to some extent, and they also served as a scaffold impregnated with bFGF.

To assess the wound tissue, we employed immunostaining. In healing tissues, fibroblasts acquire a contractile phenotype, characterized by microfilament bundle formation and de novo α-SMA expression [41]. These activated cells are termed “myofibroblasts” [42–48]. Prolonged or excessive myofibroblast activity may result in fibrosis and organ dysfunction [49–51]. One of the pigs in the PGA + bFGF group had a higher constriction rate than the other pigs, with α-SMA expression to some extent, thereby increasing the percentage of the area stained with anti-αSMA antibody. Thus, the average area occupancy rate was higher in the PGA + bFGF group than in the PGA group, but α-SMA expression was clearly suppressed in pigs that successfully received PGA + bFGF. Furthermore, similar results were obtained for calponin-1, a differentiation marker for smooth muscle cells [52], as with α-SMA. Therefore, we confirmed that PGA + bFGF can suppress myofibroblast activation.

In Elastica–Masson staining, a heavy Masson trichrome staining method, elastic fiber staining is added to Masson trichrome staining, resulting in clearly stained elastin and collagen fibers. This method showed that the mean fiber thickness was higher in the PGA and PGA + bFGF groups than that in the control group, but the difference between the thickest and thinnest areas was significantly smaller in the PGA + bFGF group than in the other groups. Therefore, in the PGA + bFGF group, the difference between sparse and dense fibroblasts was minimal. Consequently, the whole wound healed uniformly. Mihashi et al. excised the vocal cords of dogs and applied bFGF-soaked PGA sheets to the wound. As a result, the vocal cords were regenerated functionally and histologically, similar to normal tissue in terms of thickness and density [53]. Moreover, this study did not confirm the activation of the inflammatory response and immune system by the administration of PGA sheets or bFGF.

Esophageal strictures were significantly lower in the PGA + bFGF group than in the control group although the difference between the PGA + bFGF and PGA groups was not significant. In previous reports, when the scope passed, there was no passage obstruction. Assuming that the esophageal lumen is 20 mm and the scope is about 10 mm, a stenosis rate of < 50% is good, but it was < 40% only in the PGA + bFGF group.

Sealing the PGA sheets with fibrin glue containing bFGF showed no significant difference in this study. However, the procedure required no additional work other than soaking the PGA sheets in the commercialized bFGF liquid. The liquid formulation can be delivered in the same way, and obtaining additional stenosis prevention effect may be possible. Fiblast Spray costs about 8000 yen for a 500-μg bottle, making it an affordable option. The PGA sheets and fibrin used in combination also cost a total of about 40,000 yen, but the procedure is inexpensive if it does not require dozens of balloon expansions. It has been reported that 9.5% of circumferential ESD with esophageal stricture and repeated balloon dilatation did not resolve the stricture [54]. Therefore, the prevention of stenosis is essential even it is expensive. PGA sheets alone are inadequate [21], and although there was no significant difference in the stenosis rate, this rate was reduced, which is clinically significant.

This study has several limitations. First, our study had a small sample size. Second, considering that we used pigs, we cannot confirm whether our results can be easily extrapolated to human adults. Next, Fiblast Spray can only be used up to five pushes per wound for skin ulcers; thus, only eight PGA sheets of 1 cm^2^ could be sufficiently moistened. We used approximately 20 sheets; hence, 12.5 pushes of Fiblast Spray were needed. Furthermore, the package insert states that Fiblast Spray is contraindicated for cancerous areas because it promotes cell proliferation. In most cancers, cancer cell growth may be promoted by bFGF application [55–57]. It is well known that immunosuppressive conditions contribute to the development of cancer [58]. However, although it is well known that long-term administration of steroids has been shown to suppress immunity, there is no report that it contributed to the development of cancer. In particular, local steroid injections are unlikely to contribute to carcinogenesis. Even if the cancer is resected by ESD, administering bFGF before pathological evaluation for residual disease remains controversial. However, in cases where the depth of invasion is limited to the lamina propria mucosa, the current technology can diagnose the depth of invasion preoperatively with an accuracy of 90% or more [59]. Circumferential ESD is generally limited to the lamina propria mucosa. If the diagnosis is accurate, the chances of cancer remaining after ESD are low. Finally, application of PGA sheets requires a skilled technician. Mizushima et al. administered amnion-derived mesenchymal stem cells to a porcine esophageal ESD model. Thus, developing a method to deliver it in a scaffold, such as a gel, could make application easier [9].

In conclusion, stenosis after esophageal ESD is linked to fibrosis in the acute phase. Although PGA alone and PGA + bFGF showed no significant differences, the administration of PGA and bFGF suppressed myofibroblast activation in the acute phase in pigs, thereby preventing esophageal constriction.

### Supplementary Information

Below is the link to the electronic supplementary material.

Supplementary file1 (DOCX 12 kb)
