# Supplementary material for: Application of polyglycolic acid sheets and basic fibroblast growth factor to prevent esophageal stricture after endoscopic submucosal dissection in pigs
Source: J Gastroenterol. 2023 Aug 27;58(11):1094–104. doi: 10.1007/s00535-023-02032-4 (PMC10590298; doi:10.1007/s00535-023-02032-4)
Supplement: Supplementary file 1 — Supplementary file1 (DOCX 12 kb) [file 535_2023_2032_MOESM1_ESM.docx]

**Supplemental Fig. 1**

(A) Expression rate of MPO-positive neutrophils. Scale bar, 50 μm

bFGF, basic fibroblast growth factor; MPO, myeloperoxidase; PGA, polyglycolic acid

(B) Expression rate of CD107a-positive macrophages. Scale bar, 50 μm

bFGF, basic fibroblast growth factor; PGA, polyglycolic acid
